# Tracing dynamic biological processes during phase transition

**DOI:** 10.1186/1752-0509-6-S1-S12

**Published:** 2012-07-16

**Authors:** Tao Zeng, Luonan Chen

**Affiliations:** 1 Key Laboratory of Systems Biology, SIBS-Novo Nordisk Translational Research Centre for PreDiabetes, Shanghai Institutes for Biological Sciences, Chinese Academy of Sciences, Shanghai 200031, China; 2 Collaborative Research Center for Innovative Mathematical Modelling, Institute of Industrial Science, University of Tokyo, Tokyo 153-8505, Japan

## Abstract

**Background:**

Phase transition widely exists in the biological world, such as transformation of cell cycle phases, cell differentiation stages, disease development, and so on. Such a nonlinear phenomenon is considered as the conversion of a biological system from one phenotype/state to another. Studies on the molecular mechanisms of biological phase transition have attracted much attention, in particular, on different genotypes (or expression variations) in a specific phase, but with less of focus on cascade changes of genes' functions (or system state) during the phase shift or transition process. However, it is a fundamental but important mission to trace the temporal characteristics of a biological system during a specific phase transition process, which can offer clues for understanding dynamic behaviors of living organisms.

**Results:**

By overcoming the hurdles of traditional time segmentation and temporal biclustering methods, a causal process model (CPM) in the present work is proposed to study the biological phase transition in a systematic manner, i.e. first, we make gene-specific segmentation on time-course expression data by developing a new boundary gene estimation scheme, and then infer functional cascade dynamics by constructing a temporal block network. After the computational validation on synthetic data, CPM was used to analyze the well-known *Yeast *cell cycle data. It was found that the dynamics of the boundary genes are periodic and consistent with the phases of the cell cycle, and the temporal block network indeed demonstrates a meaningful cascade structure of the enriched biological functions. In addition, we further studied protein modules based on the temporal block network, which reflect temporal features in different cycles.

**Conclusions:**

All of these results demonstrate that CPM is effective and efficient comparing to traditional methods, and is able to elucidate essential regulatory mechanism of a biological system even with complicated nonlinear phase transitions.

## Introduction

In the biological world, a phase transition can be defined as the transformation of a biological system from one phenotype or state to another, where different phenotypes can be mapped to distinct states. For example, cell cycle is known to have four distinct phases: G1, S, G2 and M phases; cell differentiation contains different stages like cell proliferation, growth arrest and mature differentiation; and cancer development mainly involves three steps as mutation, promotion and invasion. Obviously, analysing those biological phase transitions will offer valuable clues for understanding life and its dynamics. Therefore, a fundamental but important question is how to trace the temporal characteristics or dynamics of a biological system during a particular phase transition process.

The study on molecular mechanism of biological phase transition has attracted much attention [1-4]. For instance, by modulating the intracellular redox state and measuring cell cycle progression, the redox cycle within the (mammalian) mouse embryonic fibroblast cell cycle was found to maintain the metabolic processes early in G1 and activate G1-regulatory proteins ahead of entry into S phase [1]. For a well known agricultural pest as migratory locust with a phase transition from the solitary to the gregarious, many down-regulated and some up-regulated genes were found in various organs when arriving to gregarious phase [2], which provides molecular indicators and recovers genetic mechanisms of phase transition in locusts. To determine the dormancy status of raspberry buds whose developmental regulation is helpful to promote the economic values of fruit and horticultural industries, a few significant dormancy-related candidate genes for raspberry buds had been identified by principal component analysis on clones' expressions [5]. Generally speaking, these research works are mainly on the different genotypes or expression variations at the level of individual genes under specific phases. Despite of those progresses, however, there is much less of focus on studying cascade changes or sequential dynamics of genes' or modules' functions at the level of networks during phase transition process.

As well known to us, one gene generally has multiple roles in biological processes but what role at a specific time is still unclear. Thus, identifying a gene functional group or module, which is composed of cooperative genes in biological processes or pathways, can reveal the functional specificity of individual genes or network modules. On the other hand, nowadays, there is rich information on biological processes [6,7], but the information on biological processes generally lacks dynamic features even compared with pathways [8,9]. Hence, in this paper we intend to identify the sequential structure or cascade dynamics of biological processes during phase transitions by developing a general framework for gene-specific segmentation and temporal block network (or network module), in particular on when and what a biological process or function will be cooperatively facilitated by network modules (or gene modules) during a phase transition. Note that, in the previous studies, the term "dynamic biological process" was usually used to refer to the dynamics of some general biological functional work-flow rather than sequential dynamics of biological processes or pathways [10-12]. In contrast, our work focuses on studying conditional and time-dependent behaviours or sequential dynamics of network modules, which are functionally enriched on specific biological processes [13].

The rapid accumulation of temporal gene expression data provides us the opportunity to unveil mechanisms of dynamic processes behind phenotype changes. In particular, a recent work shows that temporal dynamical model has ability to detect the presence and absence of stage/phase specific biological processes in *Yeast *cell cycle and metabolic cycle [13]. But, this model is limited to the analysis on the time segmentation for all genes, by simply using the replicated observations to infer biological processes' temporal coordination. To overcome this problem, a new bicluster-based temporal segmentation method in this paper is developed to build a causal process model (CPM) for identifying the temporal features of biological processes during genotype or system reorganizations. In addition to biological processes and pathways, network modules or protein complexes [14] are used to further illustrate the sequential dynamics of biological systems as the molecular basis of those functional temporal features. Actually, protein modules or protein complexes have been found to play many important roles in biological phase changes, such as, indicator of genetic effect during mammary gland oncogenesis [15], marker of cancer diagnosis and prognosis [16], predictor of genotype-phenotype associations [17,18], and responser of dynamic cues from the environment [19].

In summary, the construction of our causal process model (CPM) includes three steps. First, we identify specific biclusters with linear patterns, and assemble them into temporal blocks representing a group of genes and their time segmentations. Then, each temporal block is refined by conducting functional enrichment analysis. Finally, we infer the sequential or cascade (causal) relations between temporal blocks by a graphical model (e.g., partial correlation) among two groups of genes. Through various experiments, we demonstrate the effect of our method on gene-specific temporal segmentation. In particular, on *Yeast *cell cycle data, we show that the phase division based on CPM is more efficient and effective than the segmentation based on traditional CCC-biclustering method [20]; and in the analysis of phase/cell cycle related biological processes, we found that the group of genes actually displays conditional functional enrichment and protein interaction network rewiring. All those results show that CPM is indeed able to unveil the biological mechanism behind complicated phase transitions.

## Method

### Causal process model: temporal block based on biclusters' assembler

**Figure 1:**
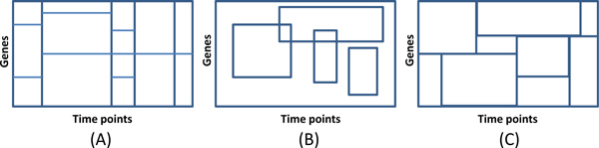
**Different schemes for time segmentation**.

Unlike traditional time segmentation methods requiring the same division on a time period for all genes [13] (see Figure 1 (A)), the gene-specific time segmentation is considered in the present work. That means, for different genes or gene groups, they can have different corresponding time segmentations based on their expressions, which can be considered as a general framework without the uniform division constraint. This is why the biclustering methodology [21,22] (see Figure 1 (B)), which can group genes and conditions simultaneously, is adopted. However, as discussed in the study of temporal dynamic model [13], state-of-the-art CCC-biclustering method [20] has the limitation that it usually cannot cover all/most genes and time points. To overcome this problem, an in-house biclustering method (noted as **EBB**: **E**rror-**B**ounded **B**iclustering) is used to enumerate so-called error-bounded linear patterns, e.g. traditional shifting pattern and scaling pattern [22], which can model a group of genes having similar expression change tendency, and further assemble them into the proposed temporal blocks by estimating the following boundary genes.

The brief framework of EBB includes three main steps: (1) discretizing the raw data matrix to a 0-1 matrix by a referred element in data matrix and a given error bound; (2) building a suffix tree based on 0-1 sequences encoded by rows in the above 0-1 matrix where '0' represents left child node and '1' represents right child node; (3) identifying the deepest right-only node in the suffix tree as a potential bicluster with error-bounded linear pattern. In fact, CCC-biclustering is also an exhaustive method [20], but it adopts a significant trend filtering to handle with the data pre-processing and thereby cannot guarantee to find all potential scaling patterns/linear patterns. This problem leads to loss of most low-signal patterns and some important expression patterns (e.g. linear patterns), which prohibit method itself to explore whole information of data. On the other hand, EBB method seeks linear patterns covering traditional shifting/scaling patterns [22] so that it can identify all interesting expression patterns in theory. Besides, EBB can also keep low-varying signals as many as possible because it uses the error bound but not the tendency bound to discrete the raw data.

As well known to us, biclusters represent similar expression behaviors of a group of genes at the same time points. However, our temporal block gathers those genes with the cooperative expression change during a specific time period, i.e. find those genes which simultaneously obtain or lose similar expression with their partner genes. Qualitatively, a temporal block is a sub-matrix in the original data to cover the complete biclusters as many as possible but split the known biclusters as few as possible. According to the following concepts and definitions, the genes on so-called temporal boundary are used to divide the whole data matrix into different matrices named as temporal blocks (see Figure 1 (C)).

**Definition 1 (Boundary gene and set) ***Given a data matrix D *= {*d*_*m,n*_}_*m*∈*I*,*n*∈*J*_*, let a set of gene expression patterns as biclusters *${\left\{P_{i}=\left\{\left(G_{i},T_{i}\right)|G_{i}\subseteq I,T_{i}\subseteq J\right\}\right\}}_{i=1}^{K}$*. Then, a gene g in I is on the temporal boundary at time point t in J only when its R value is larger than a given threshold θ with default value as one, where R is calculated as formula (1). And all boundary genes at every time point consist of a boundary set *{*BG*(*t*) = {*g*|*R*(*g*, *t*) >*θ*, *g *∈ *I*}}_*t*∈*J*_.

(1) $$R\left(g,t\right)=\frac{\left|\left\{T_{i}|g\in G_{i},t={\text{min}}_{\tau \in T_{i}}\tau \right\}\right|max\left(1,\left|\left\{\tau |\tau \in J,\tau <t\right\}\right|\right)}{\left|\left\{T_{i}|g\in G_{i},t\in T_{i},t\neq {\text{min}}_{\tau \in T_{i}}\tau \right\}\right|}$$

**Definition 2 (Temporal block) ***Given a matrix data D *= {*d*_*m,n*_}_*m*∈*I*,*n*∈*J *_*and its boundary set BG, the temporal block B_i _*= {(*G*_*i*_, *T*_*i*_)|*G*_*i *_⊆ *I*, *T*_*i *_⊆ *J*} *should satisfy following conditions:*

*(a) *$\forall g\in G_{i},g\in BG\left(\underset{\tau \in T_{i}}{\text{min}}\tau \right)$

*(b) *$\forall g\in G_{i},g\in I-BG\left(\underset{\tau \in T_{i}}{\text{min}}\tau -1\right)$*or *$\underset{\tau \in T_{i}}{\text{min}}\tau =\underset{\tau \in J}{\text{min}}\tau$

*(c) *$\forall g\in G_{i},g\in I-BG\left(\underset{\tau \in T_{i}}{\text{max}}\tau \right)$*or *$\underset{\tau \in T_{i}}{\text{max}}\tau =\underset{\tau \in J}{\text{max}}\tau$

*(d) *$\forall g\in G_{i},g\in BG\left(\underset{\tau \in T_{i}}{\text{max}}\tau +1\right)$*or *$\underset{\tau \in T_{i}}{\text{max}}\tau =\underset{\tau \in J}{\text{max}}\tau$

*(e) *∀*G *⊆ *G*_*i*_, *T *⊂ *T*_*i*_, (*G*, *T*) *does not satisfy conditions (a)-(d);*

*(f) *∀*G *⊆ *I - G*_*i*_, *T *= *T*_*i*_, (*G*, *T*) *does not satisfy conditions (a)-(d).*

For convenience, $\underset{\tau \in T_{i}}{\text{min}}\tau$ points the starting point or left-end of temporal block and $\underset{\tau \in T_{i}}{\text{max}}\tau$ points the ending point or right-end of temporal block, which are similar for temporal bicluster. Some additional differences between the proposed temporal block and traditional bicluster will be discussed in the next section.

### Causal process model: expansion of temporal block for functional enrichment analysis

**Figure 2:**
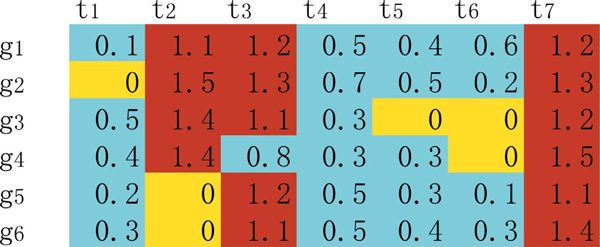
**Illustration of temporal blocks based on the estimated boundary genes**.

Like temporal segmentation, CPM gives a non-overlapping division on the whole data. It means that one gene within one time point at most belongs to one temporal block although this gene can belong to a different temporal block but at a different time, i.e. one temporal block cannot cover any other one in CPM. Taking Figure 2 as an example, six genes {(*g*_1_, *g*_2_, *g*_3_, *g*_4_, *g*_5_, *g*_6_)} might have coherent expression on time points {(*t*_3_, *t*_4_, *t*_5_, *t*_6_)}. In order to reflect the different gene reorganization events happening on time points *t*_2 _and *t*_3_, these genes are divided into two temporal blocks during the co-expression period. This is just the over-division phenomenon in biclustering study which can supply a multi-granularity model for overlapping patterns [23]. When analyzing functional enrichment on temporal blocks, the over-divided genes should be gathered again. This can be easily achieved by the expansion of temporal blocks.

**Definition 3 (Expanded temporal block) ***Given a data matrix D *= {*d*_*m,n*_}_*m*∈*I*,*n*∈*J *_*and its temporal block B*_*i *_= {*G*_*i*_, *T*_*i*_|*G*_*i *_⊆ *I*, *T*_*i *_⊆ *J*}*, the corresponding expanded temporal block *$B_{i}=\left\{G_{i},T_{i}|G_{i}\subseteq I,T_{i}\subseteq J\right\}$*satisfies: *$B_{i}^{*}=\left\{G_{i}^{*},T_{i}^{*}|G_{i}^{*}\subseteq I,G_{i}^{*}⊇G_{i},T_{i}^{*}=T_{i}\right\}$*. Where, C*_*x*,*y *_*represents the Pearson coefficient correlation between expression profiles of two genes during the time period *$T_{i}^{*}$, and p is a threshold with a default value as 0.8.

Therefore, the temporal blocks are useful to give a global scheme of the data division, and the expanded temporal blocks are suitable to reflect the local property of large data.

### Causal process model: temporal block network construction based on partial correlation

In order to extract the cascade dynamics of temporal blocks representing the sequential order of biological processes, there is a need to build a directed network among different temporal blocks whose qualitative connections are evaluated by the partial correlation [24]. It should be emphasized that, at present, our model concerns the linear relationship (i.e., linear pattern in temporal bicluster) so that the correlation but not mutual information is considered in relationship measurement. And to infer direct but not indirect correlation among genes, we adopted the partial correlation to measure association between two genes by removing the effect of their controlling genes.

**Definition 4 (Partial correlation) ***Given three gene expression profiles or vectors X,Y and Z, the partial correlation between X and Y under condition Z is calculated as:*

(2) $$PR\left(X,Y|Z\right)=\frac{C_{X,Y}-C_{X,Z}C_{Y,Z}}{\sqrt{1-C_{X,2}^{2}}\sqrt{1-C_{Y,Z}^{2}}}$$

where C.,. represents the Pearson coefficient correlation.

**Definition 5 (Link strength between temporal blocks) ***Given two temporal blocks B*_1 _= (*G*_1_, *T*_1_) *and B*_2 _= (*G*_2_, *T*_2_)*, if *$\underset{\tau \in T_{1}}{\text{min}}\tau \leq \underset{\tau \in T_{2}}{\text{min}}\tau \leq \underset{\tau \in T_{1}}{\text{max}}\tau +1$*, these two blocks have a link with direction from B*_1 _*to B*_2_*. The link strength between their referred gene expression profiles in the time period *$\left[\underset{\tau \in T_{1}}{\text{min}}\tau ,\text{min}\left(\underset{\tau \in T_{1}}{\text{max}}\tau ,\underset{\tau \in T_{2}}{\text{max}}\tau \right)\right]$*can be calculated as:*

(3) $$LS\left(B_{1},B_{2}\right)=\frac{\sum_{X\in G_{1}}{\text{max}}_{Y\in G_{2}}\left({\text{min}}_{Z\in G_{2},Z\neq X,Y}\left|PR\left(X,Y|Z\right)\right|\right)}{\left|G_{1}\right|}$$

This strength measurement indicates the potential partial relation from genes in a source block *B*_1 _to genes in a target block *B*_2_. It requires that the gene *X *in a source can directly interact with gene *Y *in a target (the correlation between X and Y is maximal as shown in the above definition), or be indirectly related to *Y *without the conduction from other target genes (the minimal partial correlation between X and Y under the control of any Z is maximal as shown in above definition). When the link strength is larger than a threshold with default value as 0.9, the connected temporal blocks are thought to have significant causal relation.

Based on the links (edges) with strengths (weights) among temporal blocks (nodes), the temporal block network (TBN) is constructed for deep analysis on dynamic biological processes. And the execution program (CPM) for temporal blocks can be accessed from http://www.sysbio.ac.cn/cb/chenlab/software.htm.

## Result and discussion

There are different characteristics between the proposed temporal blocks and traditional biclusters. Due to the module-in-focus property of biclustering, biclusters always have overlap with each other and have less size (i.e., in terms of clusters) than the original data [20]. The redundancy elimination of those overlapped biclusters is still a relevant and open question in the study of biclustering. On the other hand, in the present work, CPM suffers from few effects of potential bicluster redundancy according to the principles of temporal block construction. In order to divide original time course data, the temporal blocks instead of biclusters are used to build the dynamic model constructed by boundary gene estimation so that any temporal block is not a traditional bicluster pattern but a bicluster assembler. In other words, a temporal block does not represent the coherent expression solely as a bicluster but represents the similar expression pattern change events (condition (a) in Definition 2) as the concept of gene reorganization across the neighbouring time windows [13]. With the conditions (b), (c) and (d) in Definition 2, a temporal block can tolerate the so-called disorder period, thereby allowing the boundary genes to present at consecutive time points located at left-end of temporal block. It can also allow the so-called asynchronous ending period, i.e. allow those genes not on temporal boundary when they present at right-end of temporal block or even allow them not belonging to any original bicluster pattern. Besides, temporal blocks also have completeness guaranteed by Conditions (e) and (f) in Definition 2. These advantages of temporal blocks all let them reasonably represent the non-overlapped sub-regions of the original whole data.

For instance, in the matrix (with synthetic R values) of above Figure 2, an element in red representing its gene (row) is on the temporal boundary at its time point (column); an element in blue means that its gene is not on the temporal boundary but at the starting time point of a few biclusters; an element in orange points that its gene is not at the starting time point of any biclusters yet. Therefore, the temporal block {(*g*_5_, *g*_6_), (*t*_3_, *t*_4_, *t*_5_, *t*_6_)} is one without either disorder period or asynchronous ending period, while the temporal block {(*g*_1_, *g*_2_, *g*_3_, *g*_4_), (*t*_2_, *t*_3_, *t*_4_, *t*_5_, *t*_6_)} covers a disorder period because genes (*g*_1_, *g*_2_, *g*_3_) are at time points (*t*_2_, *t*_3_) and an asynchronous ending period for genes (*g*_3_, *g*_4_) being at time points (*t*_5_, *t*_6_).

Furthermore, the time cost of CPM is mainly on the computation of temporal block construction by temporal bicluster mining, which is similar to CCC-biclustering with a polynomial time complexity [20].

### Gene-specific temporal segmentation by CPM shown on synthetic data

**Figure 3:**
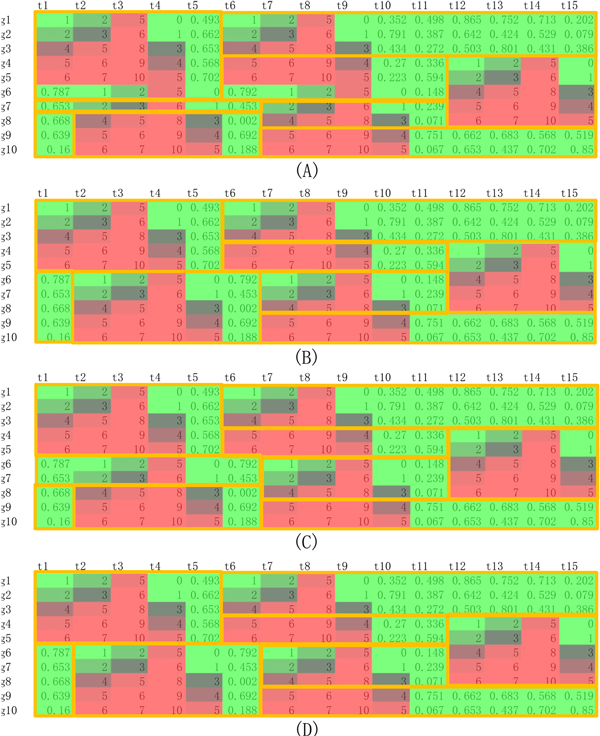
**Temporal blocks on synthetic data according to CPM with different parameter settings**.

**Figure 4:**
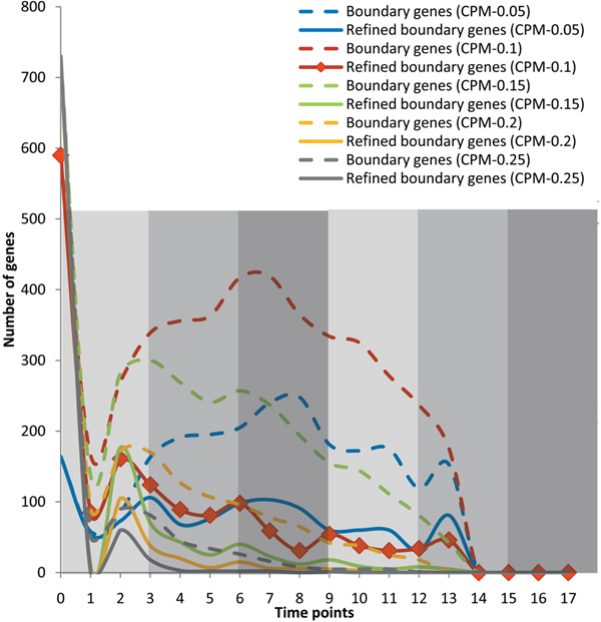
**Statistic view of boundary genes by CPM with different parameter settings**.

First of all, we analyzed CPM on a synthetic data in a simple but typical strategy adopted in the previous studies [23]. We produced a random data matrix with 10 rows and 15 columns. Five predefined blocks or patterns with five genes and four consecutive time points were embedded into such a matrix. As the recovering patterns in the above synthetic data are perfect, we used a strict error bound as 0.0001 and minimum bicluster size as 3*3,3*4,4*3,4*4 respectively to run CPM method (hereafter, the annotation x*y means that one bicluster contains at least x genes and y time points). Under different parameter settings, the divisions with temporal blocks on the whole synthetic data are shown in Figure 3, where one temporal block is surrounded by a yellow box. We should emphasize two points on these results. One is, for the effect of minimum bicluster size setting, the biclusters with a shorter time period will lead to more sub-blocks due to over-division (3*3 in Figure 3 (A) and 4*3 in Figure 3 (C)) than those with a longer time period (3*4 in Figure 3 (B) and 4*4 in Figure 3 (D)), but all blocks are still reasonable and acceptable. The other is, according to the proposed design principles, each temporal block can cover all time points of a predefined pattern and some asynchronous ending period (e.g. cases shown in Figure 3), in order to tolerate the noise/error and divide the whole data in a unified way. Totally, CPM can simultaneously group genes and find gene-specific time divisions, which cannot usually be obtained by traditional time segmentation methods, and it can further split the whole data matrix into different sub-matrices, which is disregarded in many previous biclustering studies.

### Phase description by CPM comparing with CCC-biclustering based method

Then, we analyzed CPM for the *Yeast *Cell Cycle of *α*-factor synchronization experiment of Spellman *et al. *[25]. This dataset comprises two cell cycles, with each cell cycle containing three phases as M/G1, G1&S, and G2&M [13,25]. Every phase crosses three time points in the experiment with a constant time interval as 7 minutes. After using one-way ANOVA [26] to select genes (i.e. setting the number of sample (time point) groups to be six with prior knowledge in six phases of two cell cycles, and the *P*-value to be based on the F-distribution with significant threshold as 0.05), remaining data denoted as YCC with 730 genes and 18 time points was used for further analysis. Again, we used different error bounds in {0.05, 0.1, 0.15, 0.2, 0.25} and minimum bicluster size as 10*5 (experience values in previous study) to build CPMs on YCC data for extensive evaluations.

As described before, the boundary genes can be used to trace the role-change events of a group of genes, and their number would increase greatly at a time point around the alternation of phases [13]. Due to the need to cover the possible disorder period, a few boundary genes are not effective on the temporal block construction and others are just the refined boundary genes locating at the left-end (starting time point) of final temporal blocks. According to the statistic of temporal blocks and their depending boundary genes, Figure 4 shows two kinds of distributions of boundary gene numbers under different CPM parameter settings, where the dotted line represents the distribution of the original boundary genes and the solid line represents the distribution of the refined boundary genes. Obviously, the distributions of numbers of the refined boundary genes unveil more convincible phase related characteristics than those of the original boundary genes, thereby confirming the effectiveness of the temporal blocks. Note that boundary genes mean the refined ones in the following discussions. When the error bound is strictly set to 0.05, the peaks of distributions of boundary genes are always located at the middle time of each phase because genes try to keep their status of steady coordination (note that, the strictest parameter setting as 0.01 results in no bicluster output). When error bound is set to 0.1, the peaks of distributions of boundary genes are always located at the time point of a phase transition because genes usually start to cooperatively facilitate functions at this time and temporal block can cover the potential beginning disorder period. On the other hand, when an error bound is set to 0.15 or even a larger value, distributions of boundary genes cannot keep on their correlations with phases because many noises are introduced to mix up the genes on and not on temporal boundaries. Therefore, CPM can directly use the distributions of boundary genes to trace the critical time points of phase transitions, whose dependent parameter setting will be estimated from both experience of data analyzers and pattern quality evaluation of biclustering.

**Figure 5:**
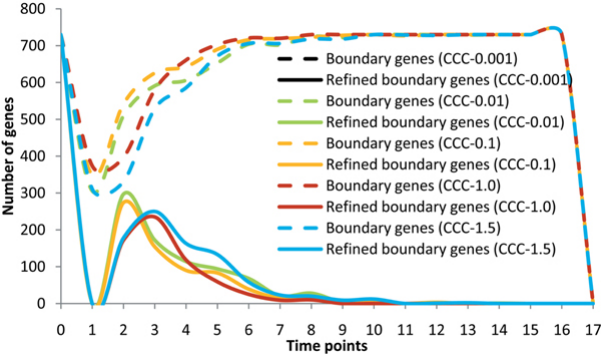
**Statistic view of boundary genes by CCC-biclustering based method with different parameter settings**.

In order to further confirm the efficiency of the proposed (EBB) bicluster-based segmentation method comparing with other bicluster-based methods, we used temporal biclusters produced by CCC-biclustering [20] (under five different parameter settings and with 1.0 as the default value) to assemble temporal blocks again and re-analyzed the relations between developmental stages and distribution of boundary genes. Compared with Figure 4, the results shown in Figure 5 illustrate that CPM is more suitable on phase description than traditional temporal biclustering based segmentation. The further discussion on the differences between bicluster-based segmentation and traditional temporal segmentation is beyond the scope of this paper because they actually belong to two distinct methodology categories like biclustering and clustering.

### Temporal trace identification by CPM with functional enrichment analysis

According to the above discussion on parameter setting, we chose the temporal blocks obtained with the most suitable error bound setting as 0.1 to conduct the following functional enrichment analysis [27]. In the temporal block expansion and temporal block network construction, the default thresholds were all used for calculation.

#### Biological processes during phase transition revealed by CPM and comparison with temporal dynamical model

**Figure 6:**
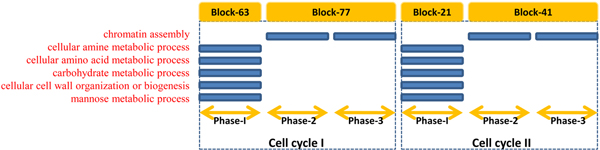
**Biological processes with potential circular behaviour enriched in phase related temporal blocks**.

Due to minimum length requirement of bicluster, the last four time points were not divided in our experiments. That is why we investigated the biological processes enriched in temporal blocks corresponding to the first phase and the latter two phases in each cell cycle, to compare with temporal dynamical model [13]. Similar to the previous studies, the circular presence and absence of some biological processes in two cell cycles are shown in a chart as Figure 6. The obtained biological processes are close to those identified by temporal dynamic model, such as amino acid biosynthetic process, cell wall chitin biosynthetic process, chromosome condensation and nucleosome assembly [13]. Therefore, CPM indeed can reveal biological processes related to phase transitions, by analyzing the phase segmentation and the temporal block network.

**Figure 7:**
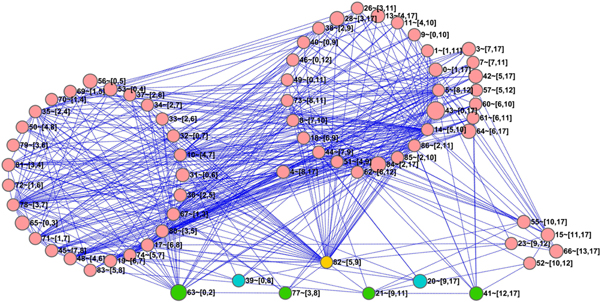
**Temporal block network on YCC dataset**.

It is worth noting that the potential causal relation between temporal blocks in CPM can further strengthen the cascade relation of phases belonging to intra- or inter-cell cycles. Figure 7 displays the whole temporal block network (where the edges between temporal blocks with same starting points were omitted so as to focus on the major asynchronous temporal relation), in which the nodes represent different temporal blocks denoted as {*TB*_*k*_}; the direct edges represent causal relations; the node label shows the id of temporal block *k *and its time segmentation [*f*, *t*] in the form as "*k *- [*f*, *t*]"; and green, blue, yellow and pink nodes mean phase related, cell cycle related, cross-phases related and other kinds of temporal blocks, respectively.

• Obviously, there are direct edges linking temporal block *TB*_63 _to *TB*_77_, and temporal block *TB*_21 _to *TB*_41_. They are actually the phases' relations belonging to intra-cell cycle, which further confirm the phase related biological processes shown in the above Figure 6.

• In all temporal blocks, only *TB*_82 _directly connects *TB*_77 _and *TB*_21_, thereby acting as a bridge of (expression) correlation between the last phases of the first cell cycle and the initial phase of the latter cell cycle. This means that CPM can also identify the phases' relations belonging to inter-cell cycle, and has the ability to infer cascade dynamics of biological functions like biological processes across multiple cell cycles. Note that, the previous temporal dynamic model needs multiple datasets to deduce causal relation between biological processes [13], however, our CPM can infer meaningful functional cascade dynamics during biological transitions even on single dataset. At present, it is actually difficult to deeply discuss the biological processes not starting at a "check point" of some phase or cell cycle due to lack of relevant biological data, however, a few processes like protein-DNA complex assembly or nucleosome assembly enriched in temporal block *TB*_82 _suggest that some of those functions will hold before entering the next phase or cell cycle.

• As the temporal dynamical model strongly shows the similarity of two cell cycles after *α*-factor handling [13], CPM can even be used to elucidate the specificities for cell cycle related temporal blocks *TB*_39 _and *TB*_20 _in Figure 7. These two cell cycle related temporal blocks (note that their functional analysis will be discussed in detail in next subsections) have not direct edges between themselves, but they can also be directly connected by temporal block *TB*_82 _again. This supports the need and importance of novel temporal blocks across neighbouring functional periods which are modelled by the gene-specific temporal segmentation integrated in CPM.

#### Functional enrichment variance during continuous cell cycles after α-factor treatment

The 1_*st *_cell cycle related temporal block *TB*_39 _covers the former three phases with time points 0-8 and has 12 genes expanded to 432 ones. On the other hand, the *2*_*nd *_cell cycle related temporal block *TB*_20 _covers the latter three phases with time points 9-17 and has 42 genes expanded to 400 ones. For those two expanded gene sets, the significant phase (cell cycle)-related biological processes and pathways are listed in Table 1 and 2. Obviously, the 1_*st *_cell cycle related genes and 2_*nd *_cell cycle related genes have shown several different biological processes annotated in GO [28], and the 1_*st *_cell cycle related genes are frequently observed in biological pathways annotated in KEGG and Reactome [29,30]. Therefore such two cell cycles after *α*-factor treatment can be just thought as two super-phases with distinct dynamical properties, which is helpful to understand the cascade dynamics of complicated biological procedures across multiple phases or cycles.

**Table 1:** Biological processes enriched in two cell cycles according to genes in *TB*_39 _and *TB*_20_

Biological process	cell 1_*st*_	cycle 2_*nd*_
mannose metabolic process	✔	
external encapsulating structure organization	✔	✔
cell wall organization or biogenesis	✔	✔
cell wall organization	✔	✔
cellular cell wall organization or biogenesis	✔	✔
cellular cell wall organization	✔	✔
cytokinetic cell separation	✔	✔
cytokinesis, completion of separation	✔	✔
cytokinesis	✔	✔
transition metal ion transport	✔	
iron ion transport	✔	
chromatin assembly	✔	✔
nucleosome assembly	✔	✔
DNA conformation change		✔
DNA packaging		✔
chromatin assembly or disassembly		✔

**Table 2:** Biological pathways enriched in two cell cycles according to genes in *TB*_39 _and *TB*_20_

Pathway	cell 1_*st*_	cycle 2_*nd*_
Amino sugar and nucleotide sugar metabolism	✔	✔
Steroid biosynthesis	✔	
Fructose and mannose metabolism	✔	
Regulation of beta-cell development	✔	
Regulation of gene expression in beta cells	✔	

**Figure 8:**
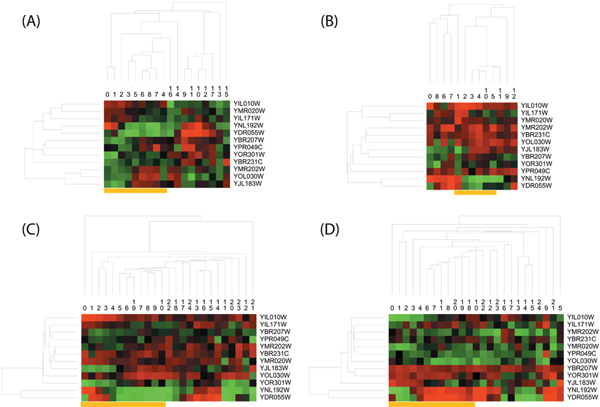
**Hierarchical clustering of genes and time points on four independent datasets according to temporal block related to 1**_***st***_** cell cycle**.

**Figure 9:**
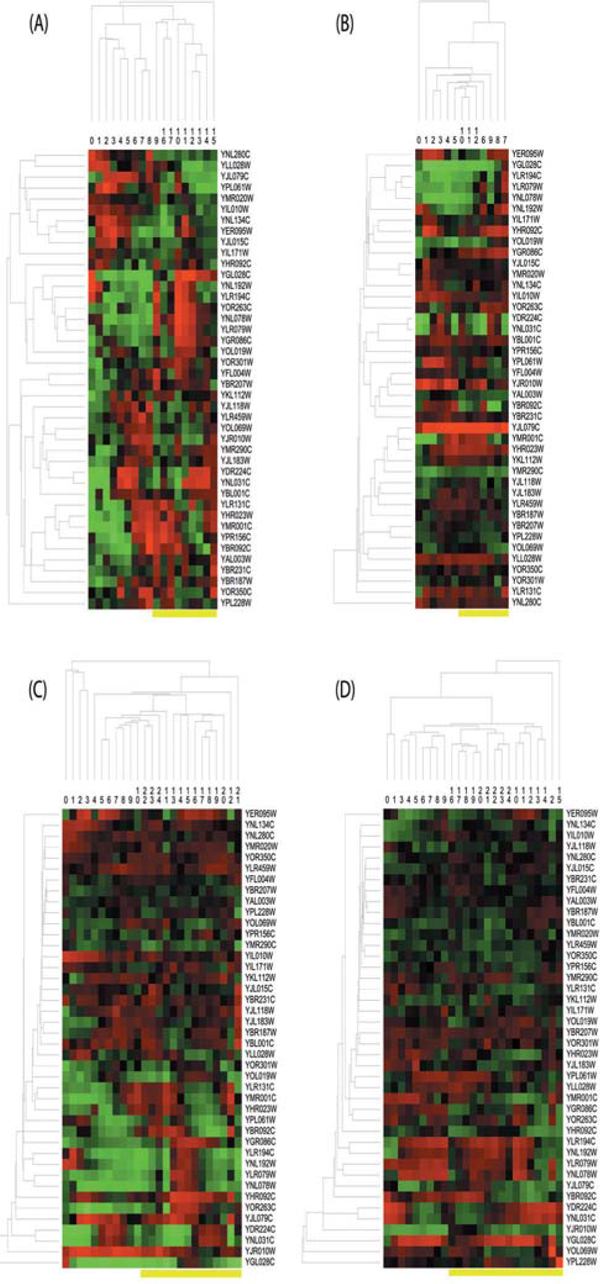
**Hierarchical clustering of genes and time points on four independent datasets according to temporal block related to 2**_***nd***_** cell cycle**.

In addition, in order to re-validate the cell cycle specificity on gene expression of such two temporal blocks, we used the genes in them to conduct hierarchical clustering with appropriate distance measurements [31] respectively on our analyzed dataset and other three independent *Yeast *gene expression datasets which also cover two cell cycles after the *α*-factor handling. They were downloaded from NCBI GEO with id GDS2318 [32] (one contributed dataset is denoted as YCC-gds2318) and GSE4987 [33] (two contributed datasets as dye-swap technical replicates are denoted as YCC-gse4987-53 and YCC-gse4987-35). On these four datasets YCC (Figure 8 (A)), YCC-gds2318 (Figure 8 (B)), YCC-gse4987-53 (Figure 8 (C)) and YCC-gse4987-35 (Figure 8 (D)) respectively, the genes from *TB*_39 _can correctly classify almost all time points into two cell cycles disregarding the effect of potential circular expression profiles in cell cycles. According to Figure 9, genes from *TB*_20 _also have good performance on clustering time points from different cell cycles. Considering the existence of missing expressions (filled with zero) of genes in other independent datasets, we only analyzed the molecular network behind such cell cycle specificity on our main YCC dataset in next subsection.

#### Relation among modules and complexes in protein interaction network rewiring and temporal trace of biological phase transitions

The co-expression network [34] was also used to reflect the potential cell cycle specificity after *α*-factor treatment through the rewired structures of the protein interaction network (PIN). Given a cell cycle related temporal block *TB*(*G*, *T*), we had a group of genes *G *and obtained the interactions of these genes' encoding proteins from STRING database [35]; with the information of *Yeast *protein subcellular localization [36] denoted as Yeast-eSLDB, we filtered the interaction by requiring that two proteins involved in one interaction must have a same candidate subcellular localization (this is because one protein may move to several subcellular localizations, and we only consider the location as "Nucleus", which has the most known protein members); based on these co-localization proteins' expression profiles in different cell cycles {*T*_*i*_}_*i*=1,2 _(for some *i*, *T *= *T*_*i*_), we calculated the Pearson coefficient correlation of two proteins with an interaction; combining the proteins and interactions with weights (or correlations), we extracted a PIN conducted co-expression network (PCCN).

**Figure 10:**
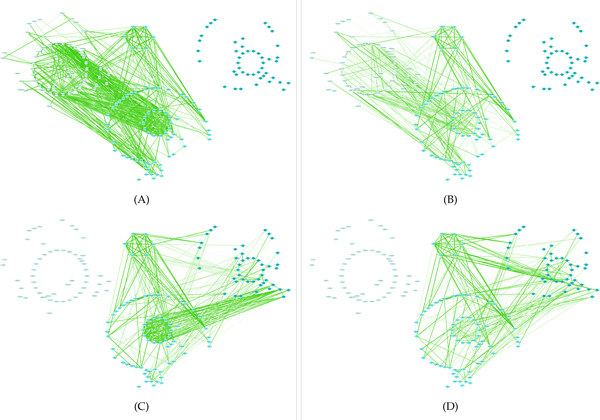
**PIN conducted co-expression networks related to genes and cell cycles corresponding to cell cycle related temporal blocks**.

Thus, we used the genes in *TB*_39 _and *TB*_20 _with their expression profiles during two cell cycles to build four PCCNs. They are denoted as ${\left\{N_{i}^{c}\right\}}_{i\in \left\{1,2\right\},c\in \left\{1,2\right\}}$, which mean that the genes/proteins in *i *cell cycle related temporal block have a rewired PCCN in actual *c *cell cycle. Figure 10(A)-(D) displays $N_{1}^{1},\;N_{1}^{2},\;N_{2}^{1}$ and $N_{2}^{2}$ respectively. As the above discussion, $N_{1}^{1}$ and $N_{2}^{2}$ should indeed have specific network characteristic corresponding to cell cycles. Generally, the genes represented by nodes in light blue belong to *TB*_39_; the genes represented by nodes in dark blue belong to *TB*_20_; while genes represented by nodes in blue belong to the overlap of such two cell cycle related temporal blocks. Each interaction edge becomes from light & thin to dark & thick when its absolute weight (or correlation) increases. By network visualization of Cytoscape [37], we easily observe the approximate network modules *C*_2 _and *C*_3 _in the Figure 10. The largest protein complex *Nucleosomal protein complex *extracted from the information of *Yeast *protein complexes [38] denoted as Yeast-CYC is also highlighted as another module *C*_1_. It is interesting that three different changes of network rewired profile correspond to the specificities of proteins in cell cycle related temporal blocks.

• For proteins in *TB*_39_, they are densely connected to module *C*_3 _in just the first cell cycle but not the second one; while *C*_3 _always has fewer contacts with proteins in *TB*_20 _so that the presence and absence of relation with module *C*_3 _would be a temporal trace for functional specificity in the first cell cycle.

• For proteins in *TB*_39 _or *TB*_20_, they present strict interactions with module *C*_2 _in the first cell cycle but lose such relation in following cell cycle. This means, in our mathematical model, *TB*_39 _mainly captures the presence of relation with *C*_2 _while *TB*_20 _tends to mine the disappearance of relation with the same module.

• Dissimilar from the above two conditions, protein complex *C*_1 _strengthens its relation with proteins in *TB*_20 _in just the second cell cycle but not the first one. Hence, the varying relation with protein complex *C*_1 _can be a candidate temporal trace for functional specificity in the second cell cycle.

Therefore, attractively, protein interaction modules and their relations with other proteins above can be thought as the dynamical markers (or temporal traces) of cell cycles in phase transitions. The proposed temporal blocks with the causal process model are indeed effective to efficiently uncover such molecular basis of a biological transition.

## Conclusion

To overcome the drawbacks of traditional time segmentation and temporal biclustering methods, the causal process model (CPM) was proposed to study the biological phase transitions in a systematic way. The experimental results validated that CPM can effectively identify gene-specific temporal segmentations by developing a boundary gene estimation scheme, and efficiently infer the potential cascade dynamics of biological processes by constructing a temporal block network. CPM not only has identified the phase-specific dynamic biological processes which were found by the traditional dynamic temporal model, but also revealed cell cycle specific rewiring of the protein interaction network which was missed in the previous studies. All in all, along with the improvement of bicluster enumeration and sparse causal network inference, the proposed CPM can both detect unknown phase transitions in real biological systems, and identify the candidate functional cascade dynamics with temporal traces (or dynamical markers) during the transformation of a biological system.

## Competing interests

The authors declare that they have no competing interests.

## Authors' contributions

TZ and LC conceived the research. TZ performed the study. LC supervised the project. TZ drafted a version of the manuscript. All authors wrote and approved the manuscript.
