# Correction: Tracing dynamic biological processes during phase transition

**DOI:** 10.1186/1752-0509-6-S1-S23

**Published:** 2012-08-23

**Authors:** Tao Zeng, Luonan Chen

**Affiliations:** 1 Key Laboratory of Systems Biology, SIBS-Novo Nordisk Translational Research Centre for PreDiabetes, Shanghai Institutes for Biological Sciences, Chinese Academy of Sciences, Shanghai 200031, China; 2 Collaborative Research Center for Innovative Mathematical Modelling, Institute of Industrial Science, University of Tokyo, Tokyo 153-8505, Japan

## Correction

After the publication of our article [1], we found that a few errors were introduced during the production process.

The mathematical definition of expanded temporal block given in Definition 3 (Expanded temporal block) should have been "the corresponding expanded temporal block $B_{i}^{*}=\left\{G_{i}^{*},T_{i}^{*}|G_{i}^{*}\subseteq I,G_{i}^{*}⊇G_{i},T_{i}^{*}=T_{i}\right\}$ satisfies: $\forall x\in G_{i}^{*},\exists y\in G_{i},s.t.\;C_{x,y}\geq p$" rather than "the corresponding expanded temporal block $B_{i}=\left\{G_{i},T_{i}|G_{i}\subseteq I,T_{i}\subseteq J\right\}$ satisfies: $B_{i}^{*}=\left\{G_{i}^{*},T_{i}^{*}|G_{i}^{*}\subseteq I,G_{i}^{*}⊇G_{i},T_{i}^{*}=T_{i}\right\}$".

And the subscript of some variable involved in partial correlation calculation (formula (2)) given in Definition 4 (Partial correlation) should have been "X, Z" rather than "X, 2". The corrected formula is shown here:

$$PR\left(X,Y|Z\right)=\frac{C_{X,Y}-C_{X,Z}C_{Y,Z}}{\sqrt{1-C_{X,Z}^{2}}\sqrt{1-C_{Y,Z}^{2}}}$$

We regret any inconvenience these errors may have caused.

## Competing interests

The authors declare that they have no competing interests.
